# Supplementary material for: Enteric ChAT‐Expressing Neurons Mediate the Protective Effect of Lactobacillus plantarum in Inflammatory Bowel Disease
Source: Adv Sci (Weinh). 2026 Oct 14:e77455. Online ahead of print. doi: 10.1002/advs.77455 (PMC13669556; doi:10.1002/advs.77455)
Supplement: Supplementary file 1 [file ADVS-9999-e77455-s001.docx]

**Supplementary Table 1. Inclusion and exclusion criteria**

**Healthy controls inclusion criteria**

Healthy controls were individuals of both sexes, ages between 18 and 65 years, Chinese nationality, without a history of inflammatory bowel disease (IBD), no relatives with IBD, no histories of autoimmune disease, immune mediated conditions, infectious colitis, and colon cancer, or a family history of colon cancer, and who were overall healthy presenting for routine screening with no other disease history.

**Patients with ulcerative colitis inclusion criteria**

- Patients with ulcerative colitis were subjects of both sexes, and 18 to 65 years old
- Patients were treatment-naive before fecal collection
- Diagnosis was in line with the active period of UC

**Exclusion criteria**

- During pregnancy or lactation
- Bleeding disorder or an acute gastrointestinal infection
- Malignancy with chemotherapy
- Presence of mental disorders
- Subjects diagnosed with indeterminate colitis, or had undergone a prior, major gastrointestinal surgery such as an ileal/colonic diversion or j-pouch
- Subjects receiving antibiotics, probiotics or other drugs within 4 weeks, following a specific diet, using prebiotics or suffering from irritable bowel syndrome (IBS) before the fecal sampling

**Supplementary Table 2.** **Clinical characteristics of UC patients and healthy controls**

| Parameters at entry | HC | UC | *P* value | Normal values |
| --- | --- | --- | --- | --- |
| Number of patients | 26 | 35 |  |  |
| Male | 13 | 18 | NS |  |
| Female | 13 | 17 | NS |  |
| Age(years) | 52.84 ± 11.24 | 46.26 ± 15.55 | NS |  |
| Laboratory data (median, IQR) |  |  |  |  |
| Hb (g/L) | 138.65 ± 17.44 | 125.46 ± 20.28 | ** | 4.3-5.8 |
| CRP(mg/L) | <0.5 | 21.12 | **** | 3.5-9.5 |
| Leukocyte(10^9/L) | 4.812±1.067 | 7.300±2.010 | **** |  |
| Neutrophil absolute value(10^9/L) | 2.851 ± 0.877 | 4.955 ± 1.822 | **** | 1.8-6.3 |
| Monocyte absolute value(10^9/L) | 0.370 ± 0.119 | 0.519 ± 0.262 | * | 0.1-0.6 |
| Lymphocyte absolute value(10^9/L) | 1.468 ± 0.414 | 1.611 ± 0.491 | NS |  |
| Basophil absolute value(10^9/L) | 0.027 ± 0.015 | 0.034±0.021 | NS |  |
| Eosinophilic absolute value(10^9/L) | 0.095 ± 0.077 | 0.180±0.174 | NS |  |
| Neutrophil ratio | 0.588 ± 0.087 | 1.567 ± 0.096 | ** |  |
| Monocyte ratio | 0.077 ± 0.019 | 0.071 ± 0.023 | NS | 0.4-0.75 |
| Lymphocyte ratio | 0.309± 0.079 | 0.231 ± 0.079 | *** | 0. 2-0.5 |
| Basophilic granulocyte ratio | 0.007± 0.006 | 0.005 ± 0.003 | NS | 1.1-3.2 |
| Eosinophilic ratio | 0.021± 0.019 | 0.025 ± 0.024 | NS | 0.00-0.06 |

Data represents mean ±SEM. NS, no statistical significance. P values were calculated by unpaired Student’s t-test.

**Supplementary Table 3. Sources of *Lactobacillus* strains**

| Strain | Source |
| --- | --- |
| *Lactobacillus plantarum* E853 | Goose's feet in foam |
| *Lactobacillus plantarum* D333 | Pickles |
| *Lactobacillus plantarum* A736 | Fermented green vegetable |
| *Lactobacillus plantarum* E374 | Soaked chicken feet |
| *Lactobacillus plantarum* A241 | Chopped pepper |
| *Lactobacillus plantarum* C347 | Sauerkraut |
| *Lactobacillus paracasei* A764 | Fermented green vegetable |
| *Lactobacillus paracasei* A7411 | Fermented green vegetable |
| *Lactobacillus paracasei* C941 | Milk fan |
| *Lactobacillus paracasei* E857 | Goose's feet in foam |
| *Lactobacillus fermentum* E451 | Pickled cucumber |
| *Lactobacillus fermentum* E452 | Pickled cucumber |
| *Lactobacillus fermentum* E464 | Pickled cucumber |
| *Lactobacillus fermentum* E465 | Pickled cucumber |
| *Lactobacillus alimentarius* A457 | Fermented green vegetable |
| *Lactobacillus alimentarius* N101 | Fermented black beans |
| *Lactobacillus alimentarius* A2801 | Pickled cowpea |
| *Lactobacillus alimentarius* D1804 | Fermented pickled cabbage |
| *Lactobacillus brevis* B651 | Chopped chili |
| *Lactobacillus brevis* C231 | Fermented allium chinense |
| *Lactobacillus brevis* D4101 | Fermented bean curd |
| *Lactobacillus delbrueckii* O171 | Fermented bean curd |
| *Lactobacillus delbrueckii* N43 | Fermented pickled cabbage |
| *Lactobacillus delbrueckii* E1705 | Soy bean |
| *Lactobacillus delbrueckii* O65 | Fermented bean curd |
| *Lactobacillus casei* J3704 | Pickled pepper |
| *Lactobacillus casei* C5603 | Pickled radish |
| *Lactobacillus casei* F3701 | Sauerkraut |
| *Lactobacillus helveticus* K0708 | Fermented radish |
| *Lactobacillus reuteri* I5202 | Skin on milk |
| *Lactobacillus nagelii* J4603 | Sweet white spirit |
| *Lactobacillus nagelii* B0802 | Puffed pear |
| *Lactobacillus nagelii* I3102 | Pickled pineapple |
| *Lactobacillus sakei* O163 | Bittern rot |
| *Lactobacillus sakei* S202 | Pickled cucumber |
| *Lactobacillus sakei* E3006 | Pickled leek flowers |
| *Lactobacillus sakei* I108 | Fermented pickled cabbage |
| *Lactobacillus sakei* E2702 | Develop cured meat |
| *Lactobacillus oryzae* I117 | Sauerkraut |
| *Lactobacillus oryzae* O1614 | Bittern rot |
| *Lactobacillus oryzae* C5306 | Sauerkraut iii |
| *Lactobacillus oryzae* C1203 | Sauerkraut |
| *Lactobacillus pentosus* C0502 | Preserved bean curd in oil |
| *Lactobacillus pentosus* E5901 | pickles |
| *Lactobacillus pentosus* D3203 | Rice wine |
| *Lactobacillus pentosus* E1004 | hand-to-hand |
| *Lactobacillus coryniformis* A7513 | Fermented green vegetable |
| *Lactobacillus coryniformis* C1207 | sauerkraut |
| *Lactobacillus coryniformis* F1702 | Fermented radish |
| *Lactobacillus coryniformis* F3005 | Fermented Chinese cabbage |

**Supplementary Table 4. Diet composition**

| Product | Control diet | | Low-Trp diet | | High-Trp diet | |
| --- | --- | --- | --- | --- | --- | --- |
|  | gm% | kcal% | gm% | kcal% | gm% | kcal% |
| Protein | 18.0% | 18.2% | 18.0% | 18.2% | 18.0% | 18.2% |
| Carbohydrate | 65.5% | 66.0% | 65.5% | 66.0% | 65.5% | 66.0% |
| Fat | 7.0% | 15.9% | 7.0% | 15.9% | 7.0% | 15.9% |
| Total |  | 100.0% |  | 100.0% |  | 100.0% |
| kcal/gm | 3.97 |  | 3.97 |  | 3.97 |  |
| Ingredient | gm | kcal | gm | kcal | gm | kcal |
| L-Arginine | 6.3 | 25.2 | 6.3 | 25.2 | 6.3 | 25.2 |
| L-Histidine | 4.5 | 18 | 4.5 | 18 | 4.5 | 18 |
| L-Lysine HCl | 16.1 | 64.4 | 16.1 | 64.4 | 16.1 | 64.4 |
| L-Tyrosine | 9.2 | 36.8 | 9.2 | 36.8 | 9.2 | 36.8 |
| L-Tryptophan | 2.1 | 8.4 | 0.21 | 0.84 | 14.7 | 58.8 |
| L-Phenylalanine | 8.7 | 34.8 | 8.7 | 34.8 | 8.7 | 34.8 |
| L-Methionine | 4.5 | 18 | 4.5 | 18 | 4.5 | 18 |
| L-Cystine | 3.7 | 14.8 | 3.7 | 14.8 | 3.7 | 14.8 |
| L-Threonine | 6.6 | 26.4 | 6.6 | 26.4 | 6.6 | 26.4 |
| L-Leucine | 15.3 | 61.2 | 15.3 | 61.2 | 15.3 | 61.2 |
| L-Isoleucine | 8.4 | 33.6 | 8.4 | 33.6 | 8.4 | 33.6 |
| L-Valine | 9.9 | 39.6 | 9.9 | 39.6 | 9.9 | 39.6 |
| Glycine | 3.1 | 12.4 | 3.1 | 12.4 | 3.1 | 12.4 |
| L-Proline | 20.4 | 81.6 | 20.4 | 81.6 | 20.4 | 81.6 |
| L-Glutamic Acid | 36.2 | 144.8 | 38.09 | 152.36 | 23.6 | 94.4 |
| L-Alanine | 4.5 | 18 | 4.5 | 18 | 4.5 | 18 |
| L-Aspartic Acid | 11.3 | 45.2 | 11.3 | 45.2 | 11.3 | 45.2 |
| L-Serine | 9.4 | 37.6 | 9.4 | 37.6 | 9.4 | 37.6 |
| Total L-Amino Acid | 180.2 | 720.8 | 180.2 | 720.8 | 180.2 | 720.8 |
|  |  |  |  |  |  |  |
| Cornstarch | 399.9 | 1599.6 | 399.9 | 1599.6 | 399.9 | 1599.6 |
| Maltodextrin | 145 | 580 | 145 | 580 | 145 | 580 |
| Sucrose | 100 | 400 | 100 | 400 | 100 | 400 |
| Cellulose | 50 | 0 | 50 | 0 | 50 | 0 |
| Soybean Oil | 70 | 630 | 70 | 630 | 70 | 630 |
| Choline Bitartrate | 2.5 | 0 | 2.5 | 0 | 2.5 | 0 |
| Mineral Mix S10022G | 35 | 0 | 35 | 0 | 35 | 0 |
| Vitamin Mix V10037 | 10 | 40 | 10 | 40 | 10 | 40 |
| Sodium Bicarbonate | 7.4 | 0 | 7.4 | 0 | 7.4 | 0 |
|  | 1000 | 3970.4 | 1000 | 3970.4 | 1000 | 3970.4 |

**FIGURE S1**

**FIGURE S2**

**FIGURE S3**

**FIGURE S4**

**FIGURE S5**

**FIGURE S6**

**FIGURE S7**

**FIGURE S1 The** **abundance of *Lactobacillus* is reduced in UC patients and DSS-induced mice, related to Figure 1.** (A) The abundance of *Lactobacillus* genus from UC patients in diverse disease course by a previous report [41]. (B) Body weight change. (C) Disease activity index (DAI). (D) Representative image of hematoxylin and eosin (H&E)-staining sections in colon. Scale bar, 100 μm. (E) Histological scores. (F) Principal co-ordinates analysis (PCoA) of stool samples based on unweighted unifrac distances by 16S rRNA sequencing. ANOSIM (analysis of similarities), R = 0.983259, p = 0.001. (G) Averaged relative abundance of bacteria at the genus level from Control and DSS. (H) Linear discriminative analysis (LDA) score of differentially enriched bacterial genera measured by LEfSe analysis between Control and DSS. (I) Relative abundance of *Lactobacillus* genus from Control and DSS in mice. (J-K) Correlation analysis of *Lactobacillus* abundance with DAI (J) and histological scores (K). Control group (Control, n=8) DSS group (DSS, n=8). Mean ± SEM shown. ns, not significant, *p < 0.05, **p < 0.01, ***p < 0.001, and ****p < 0.0001, determined by unpaired Student’s t-test.

**FIGURE S2** **Physiological characteristics of *Lactobacillus* strains in vitro, related to Figure 2.** (A-N) Growth curve of *L. plantarum* (A), *L. paracasei* (B), *L.fermentum* (C), *L. alimentarius* (D), *L. brevis* (E), *L. delbrueckii* (F), *L.casei* (G), *L.helveticus* (H), *L.reuteri* (I), *L. nagelii* (J), *L. sakei* (K), *L.oryzae* (L), *L.pentosus* (M), and *L.coryn iformis* (N) (n=3). (O) The survival rate of *Lactobacillus* strains following simulated gastrointestinal fluids. (P) Antibacterial activity. (Q) Diagram of a Caco-2 monolayer. (R) Adhesion of the *Lactobacillus* strains with Caco-2 cell monolayers (MOE 10, n=3). (S) 4 kDa FITC-dextran paracellular transport from the apical to basolateral compartment on a Caco-2 cell monolayer pretreated with *Lactobacillus* strains (MOE; 10, 24 h) before TNFα exposure (20 ng/mL, 24 h, n=3). (T) Fuzzy synthetic evaluation (FSE) analysis. (U) Loading plot of PCA. (V) Score plot of PCA based on the survival rate, antibacterial activity, adhesion and intestinal permeability of *Lactobacillus* strains in vitro. Mean ± SEM shown. Data are determined by one-way ANOVA with Tukey’s multiple comparison test. ns, not significant, *p < 0.05, **p < 0.01, ***p < 0.001, and ****p < 0.0001, compared with the Control group, ^#^p<0.05, ^##^p<0.01, ^###^p < 0.001, and ^####^p < 0.0001, compared with the TNFα group.

**FIGURE S3** ***Lactobacillus*** **strains ameliorate colitis symptoms, inflammation and mucus barrier damage, related to Figure 2.** (A) Experimental diagram for *Lactobacillus* strains administration schedule. (B) Body weight change (n=8). (C) Body weight change at day 8 after DSS induction (n=8). (D) Disease activity index (DAI) score (n=8). (E) DAI at day 8 after DSS induction (n=8). (F) Representative photographs of colon. (G) Colon length (n=8). (H and I) Representative image of hematoxylin and eosin (H&E)-staining sections in colon and histological scores (n=6). Scale bar, 100 μm. (J-K) Relative gene expression of proinflammatory cytokines including TNF-α (J) and IL-1β (K) in colonic tissues accessed by quantitative real-time PCR. (L-M) AB/PAS staining of the distal colon and elevation of goblet cells per crypt (n=3). Scale bar, 100 μm. (N-O) Representative immunostaining images and quantification of Muc2 in the colon (n=3). Scale bar, 100 μm. (P) Relative gene expression of Muc2 in colonic tissues accessed by quantitative real-time PCR. Data are determined by two-way ANOVA with Tukey’s multiple comparison test (B-E) or one-way ANOVA with Tukey’s multiple comparison test (G-O). ns, not significant, *p < 0.05, **p < 0.01, ***p < 0.001, and ****p < 0.0001, compared with the Control group, ^#^p<0.05, ^##^p<0.01, ^###^p < 0.001, and ^####^p < 0.0001, compared with the DSS group.

**FIGURE S4** ***Lactobacillus* strains ameliorate resulting epithelial barrier damage, related to Figure 2.** (A-F) Representative immunofluorescence staining images and quantification of Claudin-1, ZO1 and Occludin in the colon (n=3). Scale bar, 100 μm. (G-I) Relative gene expression of Claudin-1(G), ZO1(H) and Occludin (I) in colonic tissues accessed by quantitative real-time PCR. (J) PCA based on the body weight change, disease activity index, colon length, histological scores, goblet cells, the mRNA expression of TNF-α, IL-1β, Muc2, Claudin-1, ZO1 and Occludin in colon tissues. (K) Fuzzy synthetic evaluation (FSE) analysis. Data are determined by one-way ANOVA with Tukey’s multiple comparison test. ns, not significant, *p < 0.05, **p < 0.01, ***p < 0.001, and ****p < 0.0001, compared with the Control group, ^#^p<0.05, ^##^p<0.01, ^###^p < 0.001, and ^####^p < 0.0001, compared with the DSS group. See also Figures S2, S3 and S4, Table S1.

**FIGURE S5** ***L.* *plantarum* A736 regulates** **intestinal immune, related to Figure 2.** (A) Experimental diagram for isolation of immune cells from colonic lamina propria (cLP). (B) The gating strategies for flow cytometry analysis of monocytes, macrophages, neutrophils and dendritic cells from cLP. (C) The gating strategies for flow cytometry analysis of Th17 and Treg cells from cLP.

**FIGURE S6** ***L. plantarum* A736 impacts neuroactive signaling pathways, neurotransmitter production** **and ChAT-expressing neurons, related to Figure 3.** (A) Volcano plot displaying differentially expressed genes in colon tissues between DSS and Control (n=8). (B) Kyoto Encyclopedia of Genes and Genomes (KEGG) analysis demonstrating distinct differences in pathways between DSS and Control (n=8). (C) Gene Set Enrichment Analysis (GSEA) (n=8). (D-E) The distribution of ChAT (D) and the proportion of major enteric neurons classes in colon or ileum from mice (E) by a published study [12]. (F-G) UMAP (F) and pie chart (G) displaying the subclustering of enteric neurons or the percentage of enteric neurons classes respectively by a published study [45]. (H) Volcano plot showing differential neurotransmitters in colon tissues between DSS and Control (n=8). (I) Linear discriminative analysis (LDA) score of differentially enriched neurotransmitters from LEfSe analysis between DSS and Control (n=8). (J) Schematic of the ENS in the colon. (K) Schematic showing ChAT-Cre/tdTomato reporter mice. (L) Immunostaining of colonic sections from ChAT-Cre/tdTomato reporter mice stained for pan-neuronal marker βIII-tubulin (green) and epithelia marker EpCam1 (white) in Control and DSS group. Scale bar, 100 μm. (M) Sylgard plate with multiple pinned fragments of colonic myenteric and submucosal plexus. Control group (Control), DSS group (DSS) and *L. plantarum* A736 treatment group (*Lp* A736).

**FIGURE S7 *L.* *plantarum* A736 impacts tryptophan metabolism and its metabolite, ILA, regulates ChAT-expressing neurons and immune responses, related to Figure 4, Figure 5 and Figure 6.** (A) Volcano plot illustrating differential metabolites in feces between DSS and *Lp* A736 (n=5). (B) PCA between MRS and MRS*+Lp* A736 by untargeted metabolome (n=5). (C) Volcano plot illustrating differential metabolites between MRS and MRS*+Lp* A736 (n=5). (D) Pathway-associated QEA of differentially enriched metabolites between MRS and MRS*+Lp* A736 (n=5). (E) The phylogenetic trees of *Lp* A736 including average nucleotide identity (ANI). (F) Kyoto Encyclopedia of Genes and Genomes (KEGG) pathway enrichment analysis of the *Lp* A736 genome. (G) Concentrations of tryptophan (Trp) and indole derivatives in feces from mice between DSS and *Lp* A736 (n=6). Trp, tryptophan; TA, tryptamine; Indole; 3-Methylindole; IPYA, indole-3-pyruvic acid; IAA, indole-3-acetic acid; IA, 3-indoleacrylic acid; IAld, indole-3-carboxaldehyde; IPA, Indole-3-propionic acid. (H) Concentrations of Trp and indole derivatives in feces from mice treated with *L. plantarum* A736 or PBS during low-tryptophan diet between DSS and *Lp* A736 (n=6). (I) Trp and indole derivatives production by *L. plantarum* A736 in MRS medium (n=6). (J) Diagram showing the gene clusters for ILA production from *L. plantarum* A736. ldhA, a gene encoding 2-hydroxyacid dehydrogenase. (K) Trp and indole derivatives production by *L. plantarum* A736 in Peptone-tryptone-tryptophan medium (PTT) medium (n=6). (L) ILA levels in the feces from healthy controls and UC patients (n=35). (M) Correlative analysis of ILA with clinical parameters including C-reactive protein (CRP) values, leukocyte, monocyte and neutrophil counts in UC patients (n=35). (N) ChAT (green) and HuC/D (red) immunostaining in the colonic myenteric plexus from ChAT-iCre mice injected with rAAV-EF1α-DIO-DTA-WPRE-hGH polyA or rAAV-EF1a-DIO-EGFP-WPRE-hGH polyA. Scale bar, 36.8 μm. DSS group (DSS) and *L. plantarum* A736 treatment group (*Lp* A736) and ILA treatment group (ILA) in vivo. Low-tryptophan (Trp) diet (Trp LD), low-tryptophan (Trp) diet and *L. plantarum* A736 (Trp LD+*Lp* A736). Original culture medium (Man-Rogosa-Sharpe, MRS) and *L. plantarum* A736 culture supernatant (MRS+ *Lp* A736). Peptone-tryptone-tryptophan medium (PTT) and *L. plantarum* A736 supernatants in peptone-tryptone-tryptophan medium (PTT+ *Lp* A736). PBS-treated ChAT-iCre mice infected with AAV-encoding EGFP viruses (ChAT^AAV-EGFP^), PBS-treated ChAT-iCre mice infected with AAV-encoding DTA viruses (ChAT^AAV-DTA^). Mean ± SEM shown. Data are determined by unpaired Student’s t-test (G-I and L). ns, not significant, *p < 0.05, **p < 0.01, ***p < 0.001, and ****p < 0.0001, compared with the Control group, ^#^p<0.05, ^##^p<0.01, ^###^p < 0.001, and ^####^p < 0.0001, compared with the DSS group.
